# Peripapillary choroidal microvasculature dropout is associated with poor prognosis in optic neuritis

**DOI:** 10.1371/journal.pone.0285017

**Published:** 2023-04-27

**Authors:** Jihei Sara Lee, Sungeun Park, Sung Sik Kim, Chan Yun Kim, Wungrak Choi, Sang Yeop Lee, Hyoung Won Bae

**Affiliations:** 1 Department of Ophthalmology, Severance Hospital, Institute of Vision Research, Yonsei University College of Medicine, Seoul, South Korea; 2 Department of Ophthalmology, Yongin Severance Hospital, Yonsei University College of Medicine, Seoul, South Korea; IRCCS San Raffaele Scientific Research Institute, ITALY

## Abstract

**Purpose:**

To identify peripapillary choroidal microvasculature dropout (MvD) in eyes with optic neuritis and its association with longitudinal changes in retinal nerve fiber layer (RNFL) and ganglion cell-inner plexiform layer (GCIP) thicknesses following diagnosis.

**Methods:**

A total of 48 eyes with optic neuritis was evaluated to identify the presence of peripapillary choroidal MvD, defined as a focal capillary loss with no visible microvascular network in choroidal layer, using optical coherence tomography (OCT) angiography (OCTA). Patients were divided based on the presence of MvD. OCT and standard automated perimetry (SAP) conducted at 1, 3 and 6 months follow-up were analyzed.

**Results:**

MvD was identified in 20 of 48 eyes (41.7%) with optic neuritis. MvD was most commonly found in the temporal quadrant (85.0%), and peripapillary retinal vessel density in the temporal quadrant was significantly lower in eyes with MvD (P = 0.012). At 6 months follow-up, optic neuritis eyes with MvD showed significantly thinner GCIP in superior, superotemporal, inferior and inferotemporal sectors (P<0.05). No significant difference was noted in SAP parameters. The presence of MvD was significantly associated with thinner global GCIP thickness at 6 months follow-up (OR 0.909, 95% CI 0.833–0.992, P = 0.032).

**Conclusion:**

Optic neuritis showed peripapillary choroidal microvascular impairment in the form of MvD. MvD was associated with structural deterioration at macular GCIP. Further studies are necessary to identify the causal relationship between microvascular impairment and retinal nerve fiber layer damage in optic neuritis.

## Introduction

Peripapillary choroidal microvascular dropout (MvD), defined as a focal and complete loss of choroidal microvasculature in the deep retinal layer, was first identified in eyes with glaucoma [1]. Since then, numerous studies have demonstrated that this phenomenon has topographical correlations with glaucomatous retinal nerve fiber layer (RNFL) defect [2] and visual field (VF) defects [3]. Its presence has also been associated with generalized choroidal vessel density depression [4], faster thinning of RNFL [5, 6] and more central VF damage [7]. Some have regarded the phenomenon as a secondary vascular change while others have put forth MvD as the evidence of vascular compromise in glaucoma, in support of the ischemic theory that suggested impaired ocular perfusion as a significant cause of the disease.

The deep retinal layer microvasculature of the peripapillary area is of particular clinical interest for optic nerve diseases like glaucoma as it shares blood supply from short posterior ciliary artery with the branches supplying optic nerve head (ONH) [2, 8]. Consequently, attempts have been made to identify MvD in other optic nerve disorders and investigators have demonstrated that the loss of microvasculature is not specific to glaucomatous damage. For instance, MvD has been noted in non-arteritic ischemic optic neuropathy (NAION) [9]. MvD has also been noted in compressive optic neuropathy (CON) [10]. Comparisons to glaucomatous MvD revealed that MvD present in CON retained features different from that of glaucoma. These results suggest diverse mechanisms of MvD and possibly various roles for MvD in the development and progression of optic nerve disorders. We hypothesized that choroidal MvD is present in eyes with optic neuritis and that its association with the course of the disease may be unique to the disease. The purpose of the present study was to characterize parapapillary choroidal MvD in optic neuritis.

## Materials and methods

### Patient selection

Data were collected retrospectively from patients who were referred to Department of Ophthalmology, Severance Hospital between March 2017 and February 2021 and subsequently diagnosed with optic neuritis. The study protocol was approved by the Institutional Review Board (IRB) of Severance Hospital (IRB No. 4-2021-1393), and followed the tenets of the Declaration of Helsinki. Informed consent was waivered due to the retrospective nature of the study. A total of 48 patients were enrolled. Patients between 18 and 65 years of age, who were followed for at least 6 months were considered eligible for inclusion. Optic neuritis was diagnosed if either of the 2 criteria was satisfied [11]. 1) Acute loss of visual acuity (VA) or visual field (VF) that demonstrated 1) ocular pain on eye movement, 2) relative afferent pupillary defect (RAPD), AND 3) abnormal color vision on Ishihara color sense test) had to show EITHER abnormal optic disc swelling (by fundus examination or optical coherence tomography (OCT)), OR gadnolium-enhancement of optic nerve on magnetic resonance imaging (MRI). 2) Acute loss of VA or VF that demonstrated RAPD and abnormal color vision but not ocular pain on eye movement were required to demonstrate BOTH abnormal optic disc swelling AND gadnolium enhancement of optic nerve on MRI. All included patients had to have visual symptoms lasting less than 14 days. A total of 193 eyes from 193 consecutive patients, who were referred to our clinic for presented loss of vision with suspicious disc swelling between March 2017 and February 2021 were initially evaluated, and patients meeting any of the following criteria were excluded: 1) eyes without β-peripapillary atrophy (PPA) on fundus photographs or OCT (n = 9 eyes); 2) eyes with peripapillary γ-zone with a maximum horizontal width exceeding 200 μm on infrared imaging (n = 8); 3) refractive error of less than -8.00 D or greater than +3.00 D (n = 7); 4) significant media opacity such as cataract (n = 4); 5) clinical evidence of intracranial lesion, neurologic disorder, rheumatologic disease or systemic vasculitis (n = 16); 6) systemic medication known to induce optic neuropathy (e.g. ethambutol, digoxin and vigabatrin) (n = 13); 7) history of optic neuritis in 6 months before the initial evaluation or evidence of a new attack during 6 months of follow-up period (n = 6); 8) history of ocular trauma or intraocular surgery other than uncomplicated cataract extraction in the affected eye (n = 21); 9) presence of glaucomatous disc (i.e. neuroretinal rim thinning, notching or localized RNFL defect), history of glaucoma diagnosis prior to or following diagnosis of optic neuritis or family history of glaucoma (n = 18); 10) intraocular pressure (IOP) exceeding 21 mmHg at any point during the 6 months of follow-up (n = 4); 11) evidence of intraocular disease (optic disc abnormalities including optic disc drusen, optic disc neuroretinal rim pallor, and retinal diseases such as retinal vessel occlusion or diabetic retinopathy) (n = 21); 12) unable to rule out non-arteritic ischemic optic neuropathy (NAION) at initial presentation, including those having a fellow eye with an optic disc showing crowded morphologic appearance (“disc at risk”) (n = 10); 13) those whose diagnosis were subsequently changed to ischemic, compressive, hereditary or toxic optic neuropathy (n = 5); and 14) suspected infectious, granulomatous (e.g. sarcoidosis) and neoplastic causes according to serologic, microbiologic and radiologic tests (n = 3). Patients with a diagnosis of multiple sclerosis (MS), myelin-ligodendrocyte glycoprotein antibody-associated disease (MOGAD) or neuromyelitis optica spectrum disorder (NMOSD) were also included. Of note, MS diagnosis followed the McDonald criteria [12] while NMOSD diagnosis followed the revised diagnostic criteria [13]. MOGAD was diagnosed if optic neuritis or transverse myelitis was present with positive serological finding of anti-myelin-oligodendrocyte glycoprotein (MOG) antibody [14]. The appearance of optic disc was evaluated by 3 glaucoma specialists (H.W.B., C.Y.K., and W.C.) masked to patient information.

### Ophthalmologic evaluation

Participants underwent complete ophthalmologic evaluation during their initial visit. The examination included measurements of VA, refraction error, IOP with Goldmann applanation tonometer (GAT; Haag-Streit model BQ-900; Haag-Streit, Inc., Bern, Switzerland), and axial lengths, slit lamp examinations and dilated fundus examinations with a 90D lens. Patients also underwent color disc stereophotography, red-free fundus photography, standard automated perimetry (SAP; 24–2 SITA standard, Humphrey Field Analyzer II; Carl Zeiss Meditec, Inc., Dublin, California, USA), cirrus OCT (Carl Zeiss Meditec, Inc., Dublin, California, USA) and cirrus OCT angiography (AngioPlex; Carl Zeiss Meditec). Medical history was reviewed. Patients who were clinically diagnosed with optic neuritis subsequently underwent MRI using gadnolium contrast and serologic testing. Patients, who in the opinion of their treating physicians warranted treatment, were admitted to receive 1000mg of intravenous methylprednisolone sodium succinate daily for 3 days, followed by tapering with oral prednisolone starting from 1mg/kg body weight (maximum of 60 mg oral prednisolone). Blood pressure was measured at admission. Patients were followed at 1, 3, and 6 months since diagnosis at an out-patient clinic.

### Serological testing

Serum samples were collected following diagnosis of optic neuritis during initial visit, prior to the initiation of therapy, as per routine protocol. Complete blood cell counts, including estimated sedimentation rate (ESR), and C-reactive protein (CRP) measurements were obtained. All patients were tested for serum anti-aquaporin-4 (AQP4) antibody, anti-MOG antibody, and anti-nuclear antibodies (ANA). The presence of serum anti-AQP4 IgG antibody was evaluated with a commercially available cell-based indirect immunofluorescence assay kit (Euroimmun Medical Laboratory Diagnostics Stock Company, Germany). Intensity score 1+ or higher was considered as positive for the antibody. In-house live-cell based assay was conducted at National Cancer Center to confirm the presence of anti-MOG antibody. Briefly, HEK293T cells transfected with MOG were incubated with 1:20 diluted peripheral blood serum at room temperature for 1 hour; then, anti-human IgG1 Alexa 499 antibody at 1:750 dilution was added. After washing and cell mounting, samples were visualized under a fluorescent microscope (Nikon, Japan) and fluorescence intensity scores of 2+ or greater were considered positive. ANA was assayed by applying patient serum samples to cultured Hep-2 cells. NOVA Lite DAPI ANA Kit (Inova Diagnostics, San Diego, California, USA) was used according to the manufacturer’s specifications.

### Optical coherence tomography

Spectral domain (SD)-OCT was conducted for measurement of RNFL and ganglion cell-inner plexiform (GCIP) layer thicknesses, following OSCAR-IB and APOSTEL guidelines [15, 16]. Pupils were dilated and Cirrus Fast-Track eye-tracking technology of software version 6.0 (Cirrus OCT, Carl Zeiss Meditec, Inc., Dublin, California, USA) was used by an experienced operator to obtain optic disc 200 x 200 cube and macula 512 x 128 cube scans on the same day as other ophthalmologic evaluation. Automated segmentation was used. Thicknesses of peripapillary RNFL and macular GCIP were recorded at initial evaluation, and at 1, 3, 6 months of follow-up. All OCT scans were evaluated for image quality by a single investigator (J.S.L.) and those scans with a signal strength less than 6 or with any artifacts were excluded from analysis (2 scans at initial evaluation, 5 at 1 month, 6 at 3 months and 5 at 6 months). Incorrect automated segmentation was corrected manually by a single investigator (J.S.L.), masked to the identity of the scans.

### Optical coherence tomography angiography

The peripapillary and macular areas were imaged using a commercially available OCT angiography device (AngioPlex; Carl Zeiss Meditec). The angiography algorithm is described in detail elsewhere [17]. Briefly, the angiography device generated 350 A-scans per B-scan along the horizontal dimension and repeated the process for 350 B-scans to produce en face microvascular images. The AngioPlex software calculated vessel density (VD) by adding the total length of perfused vasculature per unit area of a 6 mm-diameter circle, and VD was evaluated in en face images of peripapillary and macular inner-retina layer, as automatically segmented by the OCT instrument software. Any segmentation error was corrected manually. All scans were examined by a single experienced grader for quality (J.S.L.) and eyes were excluded from analysis if scans had motion artifacts or low signal strength (<7/10).

### Determination of choroidal microvascular dropout

**Fig 1:**
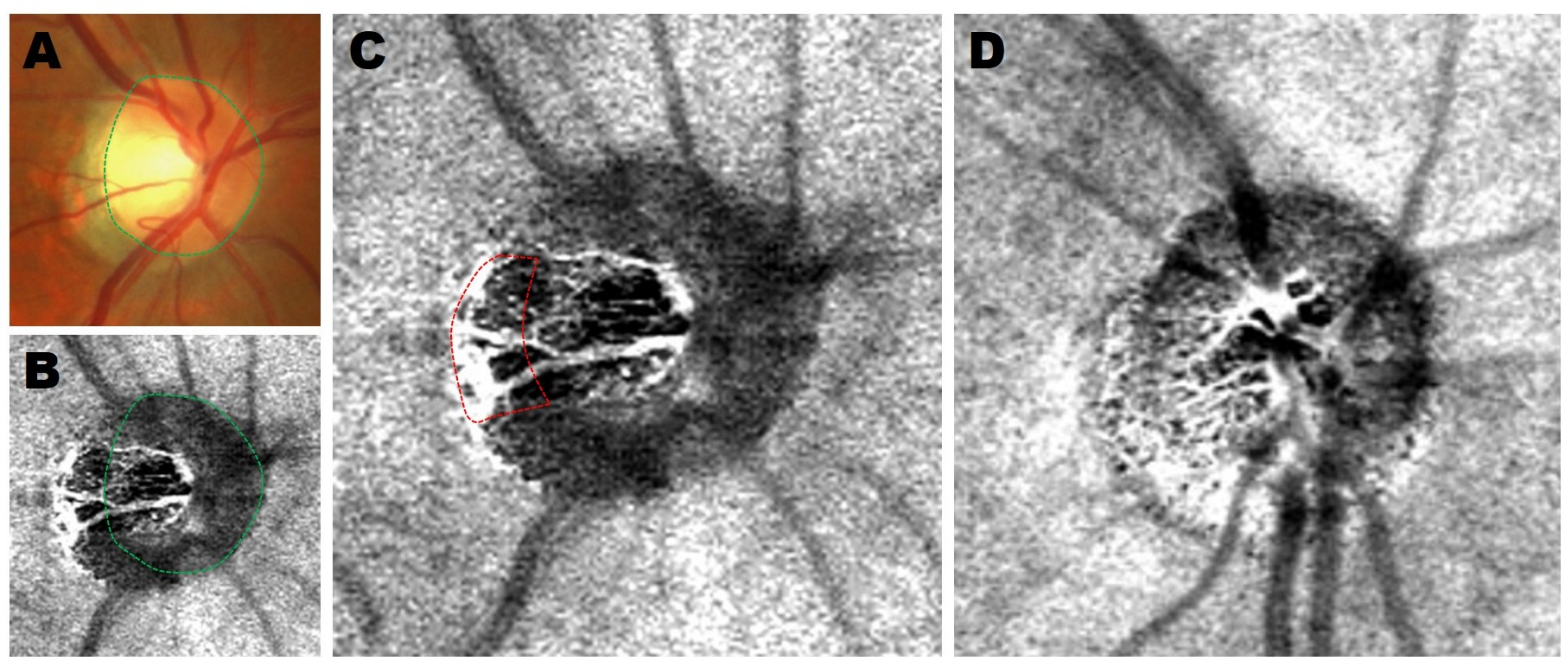
Determination of choroidal MvD. Color disc photograph (A), OCT angiography image of the disc in the deep layer (B), and magnified OCT angiography image (C) are shown. The optic disc margin is delineated with a green dashed line and the area of MvD is demarcated by a red dashed line. A disc without a choroidal MvD is shown in (D).

A microvasculature dropout (MvD) was defined as a complete focal loss of choriocapillaris or microvasculature within β-PPA on en face images of the choroid (Fig 1). The choroidal layer was visualized by manually generating an en face slab that extends from the retinal pigment epithelium to 390 μm below, a distance considered sufficient to include the choroid and the inner scleral layer [10]. A microvasculature loss of circumferential width greater than a half clock-hour of the disc circumference adjoining the disc margin was considered a MvD [18]. The location of MvD was determined based on the 4 circumferential quadrants used in the sectoral RNFL thickness evaluation of the cirrus OCT system. If an MvD spanned over more than 1 neighboring sector, the sector that included a larger portion was assigned. The MvD was identified by 2 independent observers (J.S.L and S.P.) blinded to the clinical information of patients. Any discrepancy was resolved by a third adjudicator (H.W.B.). The presence of MvD was determined at initial presentation if possible. When swollen optic discs made detection difficult, patients were reassessed at 3 months. Those who continued to show optic disc swelling were assessed again 6 months after initial presentation. Based on the presence of peripapillary choroidal MvD, optic neuritis eyes were divided into MvD+ and MvD- groups.

### Statistical analysis

Interobserver agreement on the presence of MvD was analyzed using the κ statistics. The distribution of data was identified with Wilk-Shapiro test. Continuous data were presented as mean± standard deviation (SD), and categorical data were presented as number (percentage of the group). Comparisons between the MvD+ and MvD- groups were made using independent samples t-test for normally distributed continuous variables, Mann-Whitney U test for non-normally distributed continuous variables and Chi-square test for categorical variables. Aligned rank transform (ART) analysis of variance (ANOVA) was used to compare repeated measures over time between the 2 groups. Univariate binary logistic regression analyses were performed separately for each variable. In order to build a multivariate model, a stepwise selection method was adopted with the entry P-value of <0.2 and a stay P value of <0.05. Adjusted odds ratio (OR) with 95% confidence interval (CI) were recorded. A P value <0.05 was considered statistically significant. Due to the exploratory nature of the study, no adjustments were made for multiple comparisons. All statistical analyses were performed using SPSS version 23.0 (SPSS Inc., Chicago, Illinois, USA).

## Results

### Baseline characteristics

The results of comparisons of baseline characteristics between optic neuritis patients with and without choroidal MvD are presented in Table 1. Peripapillary choroidal MvD was detected in 20 out of 48 eyes with optic neuritis (41.7%; designated as MvD+ group). The κ coefficient for interobserver agreement on the presence of MvD was 0.921. The MvD+ group was 41.2±20.1 years old and the MvD- group was 43.2±14.5 years old (P = 0.623). Patients were followed for 17.6±3.5 months and the difference in the follow-up duration was not significant (P = 0.075). The proportions of males were comparable between the two groups (P = 0.475). No significant difference was found in axial length and IOP at initial presentation. The number of errors on color sense test was also comparable. No difference was found in the prevalence of systemic conditions. Serological parameters evaluated at initial presentation, such as CRP and ESR, showed no significant differences between the 2 groups. The frequency of detection of antibodies associated with optic neuritis was also comparable. Of 20 patients belonging to the MvD+ group, 2 patients (10.0%) were subsequently diagnosed with NMOSD. In the MvD- group, 3 patients (10.7%; P = 0.660) were diagnosed with NMOSD, and 2 (7.1%; P = 0.335) were diagnosed with MS. A total of 8 patients (4 in MvD+ group, 20.0%; 4 in MvD- group, 14.3%; P = 0.442) had previously experienced optic neuritis in the same eye (at least 6 months prior to the initial evaluation).

**Table 1:** Comparison of demographic and ocular characteristics.

	MvD+	MvD-	P
	(n = 20)	(n = 28)	
Age, years	41.2±20.1	43.2±14.5	0.623^†^
Male, n (%)	6 (30.0)	7 (25.0)	0.475
Ocular variables			
Spherical error, D	-2.4±3.9	-1.2±2.2	0.557^†^
Axial length, mm	24.5±2.1	23.9±1.3	0.777^†^
IOP, mmHg	15.6±3.7	14.4±2.6	0.187*
MOPP, mmHg	45.4±8.3	45.3±10.9	0.973*
Pseudophakia, n (%)	2 (10.0)	1 (3.6)	0.373
Color sense test, no error	11.6±11.5	12.1±10.9	0.894*
RAPD, n (%)	17 (85.0)	24 (85.7)	0.628
Moving pain, n (%)	9 (45.0)	13 (46.4)	0.578
Enhancement on MRI, n (%)	12 (60.0)	19 (67.9)	0.398
Systemic diseases, n (%)			
HTN	5 (25.0)	2 (7.1)	0.083
DM	2 (10.0)	1 (3.6)	0.355
CVA	0 (0.0)	0 (0.0)	-
Multiple sclerosis	0 (0.0)	2 (7.1)	0.335
NMOSD	2 (10.0)	3 (10.7)	0.660
BMI, kg/m^2^	24.3±5.1	23.0±3.2	0.426^†^
SBP, mmHg	122.5±8.6	122.9±16.1	0.937*
DBP, mmHg	75.5±12.0	76.0±12.2	0.924*
MAP, mmHg	91.2±9.9	89.6±15.0	0.745*
Serological parameters			
CRP, mg/L	1.8±3.2	1.5±3.0	0.958^†^
ESR, mm/hr	17.2±17.1	18.3±22.4	0.811^†^
Serum antibody, n (%)			
Anti-MOG antibody	1 (5.0)	2 (7.1)	0.730
Anti-AQP4 antibody	2 (10.0)	2 (7.1)	0.520
ANA	5 (25.0)	7 (25.0)	0.637
Recurrence, n (%)	4 (20.0)	4 (14.3)	0.442
IV methylprednisolone, n (%)	18 (90.0)	24 (85.7)	0.508
Duration of symptoms, days	8.4±5.1	7.2±4.5	0.407*
Follow-up duration, months	22.9±4.1	13.9±2.7	0.075^†^

P-value <0.05 was considered statistically significant.

*P <0.05 by Student’s t-test

^†^P<0.05 by Mann-Whitney U test

Abbreviations: MvD, microvasculature dropout; IOP, intraocular pressure; MOPP, mean ocular perfusion pressure; RAPD, relative afferent pupillary defect; HTN, hypertension; DM, diabetes mellitus; CVA, cerebrovascular accident; NMOSD, neuromyelitis optica spectrum disorder; BMI, body mass index; SBP, systolic blood pressure; DBP, diastolic blood pressure; MAP, mean arterial pressure; CRP, C-reactive protein; ESR, erythrocyte sedimentation rate; MOG, myelin oligodendrocyte glycoprotein; AQP4, aquaporin-4; ANA, anti-nuclear antibody; IV, intravenous.

### Distribution of choroidal MvD and retinal vessel density at diagnosis

**Fig 2:**
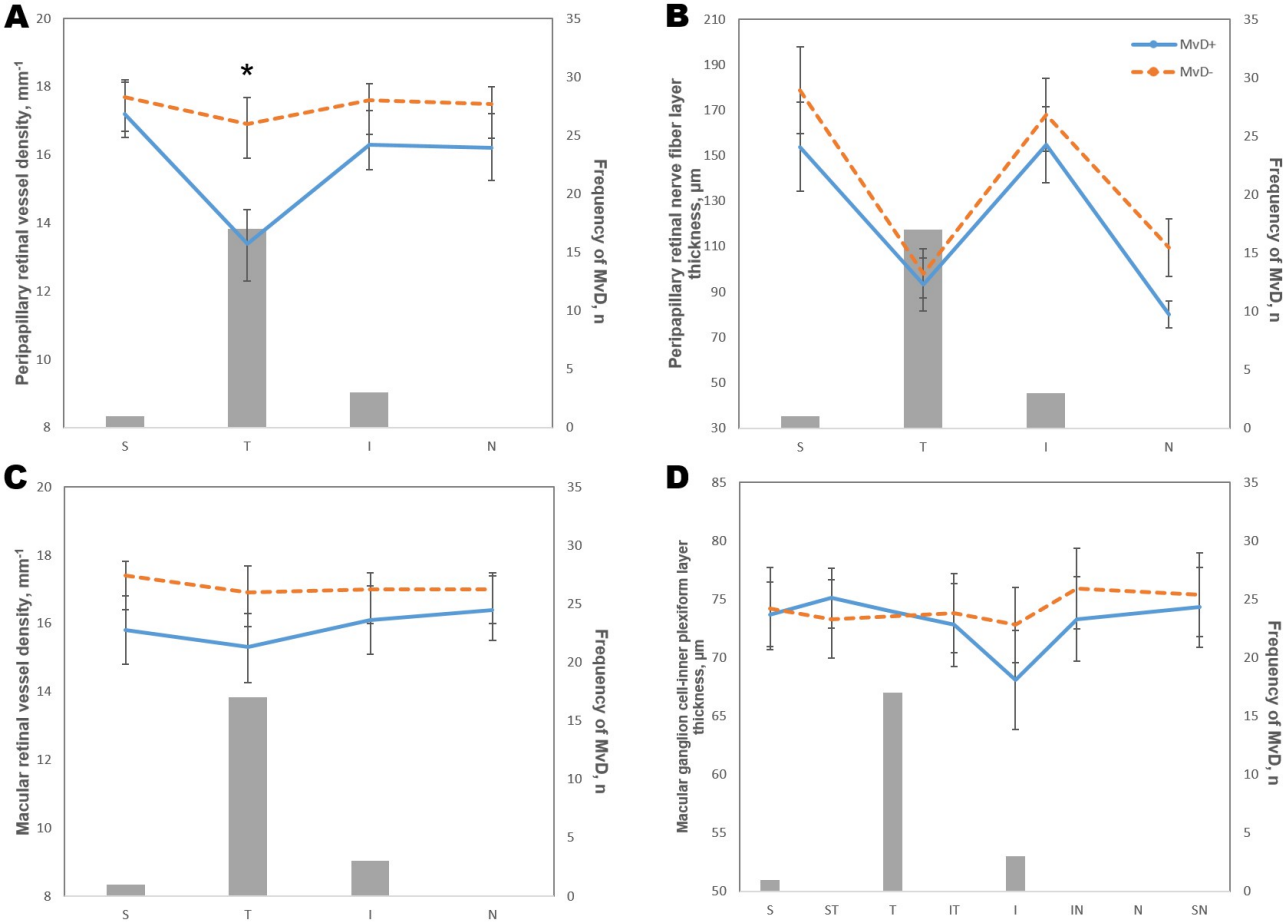
Graphs (line plots) showing initial peripapillary retinal vessel density (A), RNFL thickness (B), macular vessel density (C), and GCIP layer thickness (D) in optic neuritis eyes with and without MvD along with frequency distribution of MvD (bar plots). Choroidal MvD was most frequently found in the temporal quadrant. Peripapillary superficial retinal vessel density was significantly lower in the temporal quadrant in patients with MvD in comparison to those without MvD. An asterisk indicates a between-group difference with a P value <0.05. Error bars indicate standard errors. Abbreviations: MvD, microvasculature dropout; S, superior quadrant; T, temporal quadrant; I, inferior quadrant; N, nasal quadrant.

Choroidal MvD was most frequently found in the temporal quadrant (17/20, 85.0%). Two were detected in the inferior quadrant and the remaining 1 was detected in the superior quadrant (Fig 2). RNFL thickness at initial presentation was comparable between the MvD+ and MvD- groups whether it be global or sectoral (S1 Table). No significant difference was noted in GCIP layer thickness at initial presentation, either. Evaluation of OCTA showed that the average superficial retinal vessel density of the MvD+ group was significantly lower in the inner temporal quadrant around the optic disc (16.9±4.2 mm^-1^ vs. 13.5±5.1 mm^-1^, P = 0.016). Vessel density in the macula was not significantly different between the two groups.

### Longitudinal changes in RNFL thickness, GCIP thickness and VF parameters

**Fig 3:**
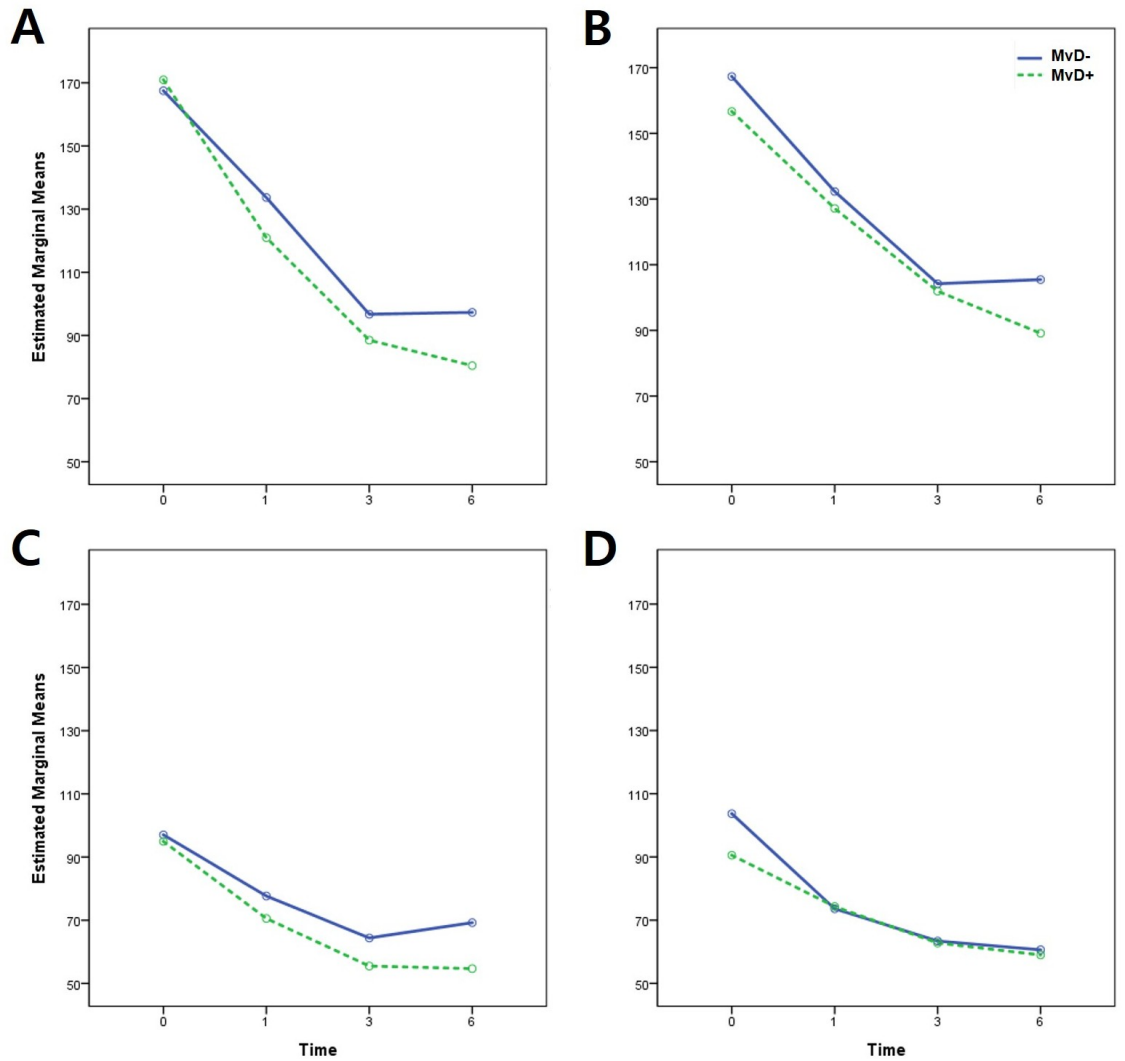
Longitudinal changes in estimated marginal means of RNFL thickness in optic neuritis eyes based on the presence of MvD in superior (A), inferior (B), temporal (C), and nasal (D) quadrants. No significant difference was noted in RNFL thickness in any quadrant 6 months after diagnosis of optic neuritis in patients displaying peripapillary choroidal MvD. Error bars indicate standard errors. Abbreviations: RNFL, retinal nerve fiber layer; MvD, microvasculature dropout.

**Fig 4:**
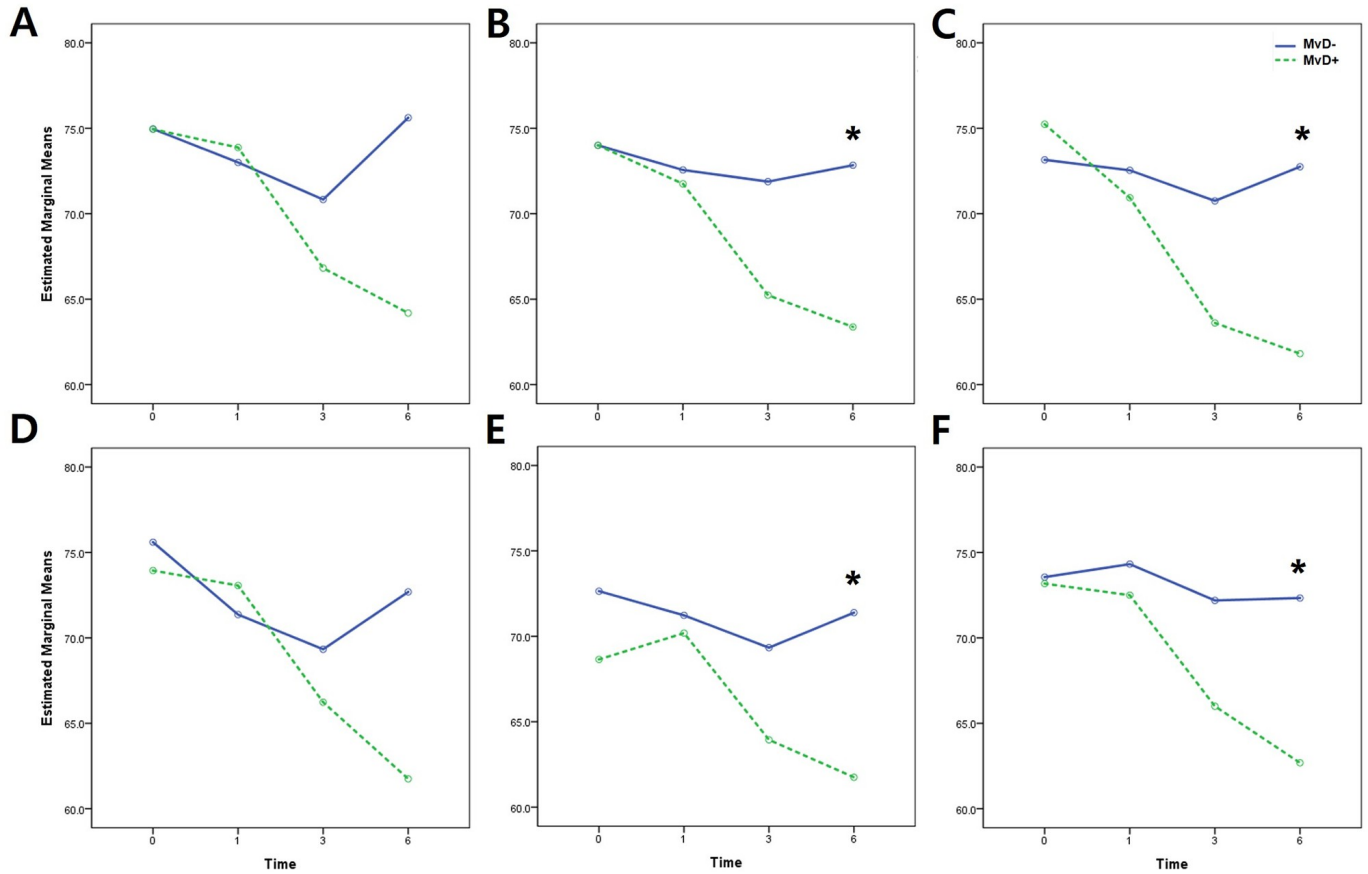
Longitudinal changes in estimated marginal means of GCIP thickness in optic neuritis eyes based on the presence of MvD in superonasal (A), superior (B), superotemporal (C), inferonasal (D), inferior (E), and inferotemporal (F) sectors. GCIP thickness was significantly lower in optic neuritis eyes displaying MvD in 4 of 6 sectors at 6 months follow-up. An asterisk indicates a between-group difference with a P value <0.05. Error bars indicate standard errors. Abbreviations: GCIP, ganglion cell-inner plexiform layer; MvD, microvasculature dropout.

Longitudinal changes in RNFL thickness were analyzed using ART ANOVA (Fig 3). Both MvD+ and MvD- groups showed a decrease in RNFL thickness over time in general, and the thicknesses were comparable until 3 months follow-up in all quadrants. At 6 months follow-up, however, the RNFL thickness of MvD+ group continued to decrease while that in the MvD- group tended to plateau, but the difference was not significant between the 2 groups in any quadrant. As for GCIP thickness (Fig 4), the GCIP thickness was significantly lower at 6 months follow-up in the MvD+ group in all superior, superotemporal, temporal, inferotemporal, and inferior sectors (P = 0.046 superior; P = 0.030 superotemporal; P = 0.037 inferotemporal; P = 0.027 inferior). When VF tests were analyzed to identify whether structural changes were also reflected in the functional parameters during the same period (S2 Table), even though the MvD+ group showed worse MD, PSD and VFI at all time points in comparison to the MvD- group, the difference was not statistically significant.

### Factors associated with choroidal MvD

**Fig 5:**
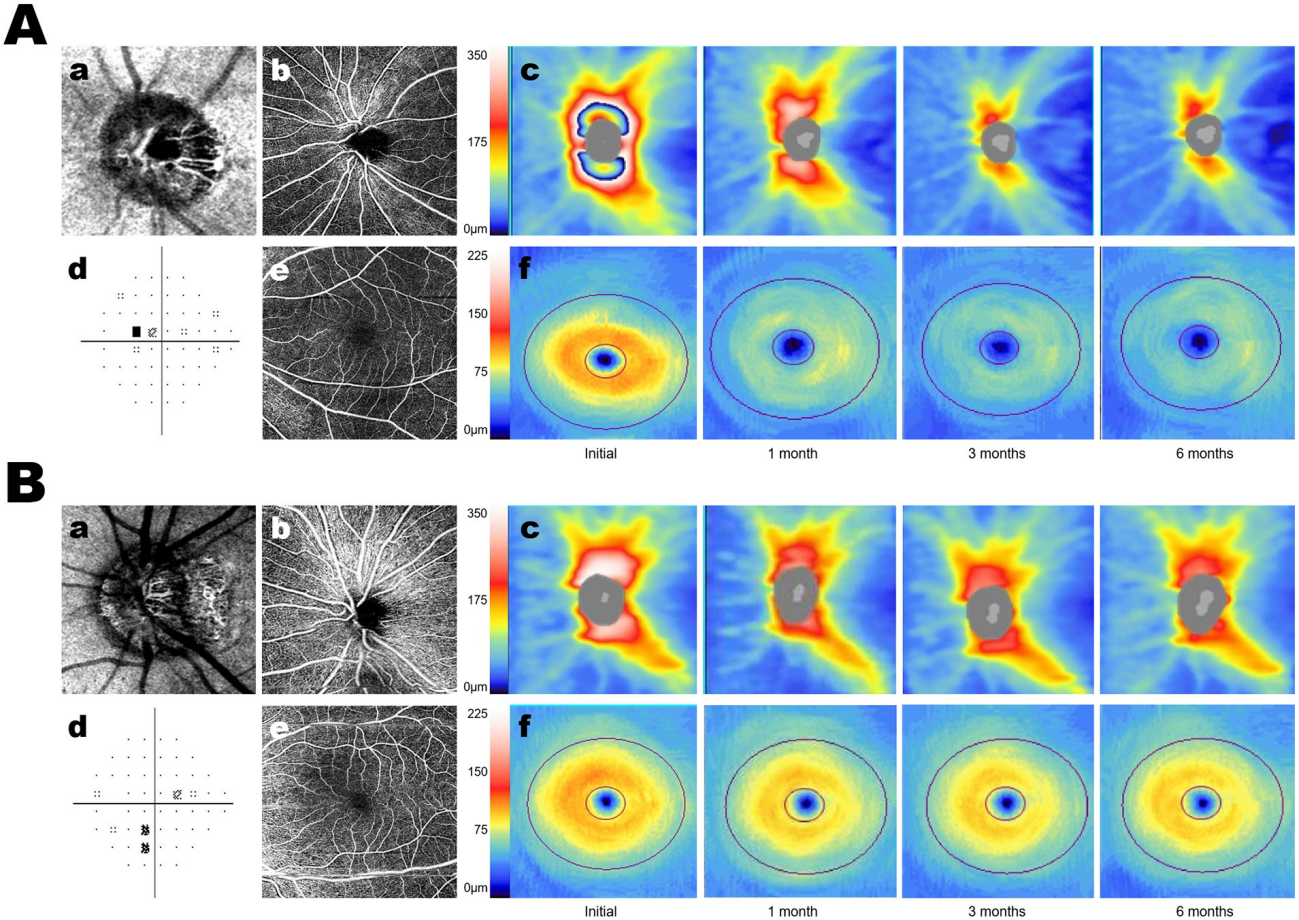
Representative cases of optic neuritis patients depending on the presence of choroidal MvD. A representative patient with optic neuritis in the left eye, along with peripapillary choroidal microvasculature dropout in the temporal quadrant (A). En-face OCT angiography images of the choroidal layer (a) and inner retinal layer (b) of the peripapillary area are shown. Visual field pattern deviation map at 6 months (d) and en-face OCT angiography image of the inner retinal layer of macula (e). Following attack of optic neuritis, RNFL thickness as shown c, and GCIP thickness as shown in F significantly decreased. A patient with optic neuritis in the left eye, without notable peripapillary choroidal microvasculature dropout in the en-face OCT angiography image of the choroidal layer (B, a). En-face OCT angiography images of the inner retinal layer of the peripapillary area (b) and the macular area (e) are shown. Visual field at 6 months showed no significant defect (d). The RNFL thickness (c) and GCIP thickness (f) showed mild decreases following the attack.

Logistic regression analyses were performed to identify factors associated with the presence of MvD in eyes with optic neuritis, as shown in Table 2. Thin RNFL and GCIP at 6 months were found to be significantly associated with the presence of MvD in univariate analyses. IOP, HTN, follow-up duration, peripapillary and macular rVD, and VF MD were carried forward as covariates to multivariate analyses, which were conducted separately for final RNFLT, final GCICPLT, initial VF MD and final VF MD to avoid collinearity. According to the results of multivariate analyses, the presence of choroidal MvD in patients with optic neuritis was associated with reduced GCIP thickness at 6 months follow-up (OR 0.909, 95% CI 0.833–0.992, P = 0.032). Representative cases are shown in Fig 5.

**Table 2:** Logistic regression analyses to identify factors associated with the presence of choroidal MvD in eyes with optic neuritis.

	Univariate analysis		Multivariate analysis 1		Multivariate analysis 2		Multivariate analysis 3		Multivariate analysis 4	
	OR (95% CI)	P	OR (95% CI)	P	OR (95% CI)	P	OR (95% CI)	P	OR (95% CI)	P
Age	0.993 (0.959–1.028)	0.690								
Male	1.286 (0.356–4.639)	0.701								
Axial length	1.282 (0.598–2.746)	0.523								
IOP	1.143 (0.937–1.395)	0.186	0.861 (0.528–1.261)	0.361	0.892 (0.569–1.396)	0.616	1.109 (0.851–1.444)	0.445	0.843 (0.448–1.586)	0.596
HTN	4.643 (0.796–27.090)	0.088	1.732 (0.106–28.235)	0.700	0.651 (0.033–8.368)	0.651	0.378 (0.045–3.175)	0.370	2.939 (0.033–263.494)	0.638
DM	3.176 (0.267–37.779)	0.360								
Peripapillary rVD	0.818 (0.642–1.043)	0.106	0.666 (0.306–1.451)	0.306	0.749 (0.382–1.471)	0.402	0.866 (0.620–1.210)	0.399	0.826 (0.227–3.011)	0.772
Macular rVD	0.837 (0.672–1.044)	0.115	0.923 (0.615–1.385)	0.698	0.866 (0.570–1.315)	0.499	0.899 (0.674–1.199)	0.469	0.653 (0.315–1.351)	0.250
Initial RNFLT	0.994 (0.984–1.005)	0.316								
Final RNFLT	0.942 (0.891–0.997)	0.038	0.921 (0.844–1.005)	0.063						
Initial GCIPT	0.991 (0.961–1.022)	0.576								
Final GCIPT	0.915 (0.858–0.976)	0.007			**0.909 (0.833–0.992)**	**0.032**				
Initial VF MD	0.957 (0.912–1.005)	0.082					0.964 (0.910–1.020)	0.203		
Final VF MD	0.915 (0.810–1.034)	0.154							0.939 (0.814–1.082)	0.384
Follow-up duration	1.035 (0.998–1.075)	0.066	1.029 (0.948–1.118)	0.493	1.032 (0.949–1.122)	0.460	1.042 (0.992–1.094)	0.103	1.028 (0.913–1.158)	0.649

P-value <0.05 was considered statistically significant.

Abbreviations: OR, odds ratio; CI, confidence interval; IOP, intraocular pressure; HTN, hypertension; DM, diabetes mellitus; rVD, retinal vessel density; RNFLT, retinal nerve fiber layer thickness; GCIPT, ganglion cell-inner plexiform layer thickness; VF, visual field; MD, mean deviation.

## Discussion

Our analyses revealed that peripapillary choroidal MvD was present in some of patients with optic neuritis. The locations of choroidal MvD tended to correspond to locations at which peripapillary retinal vessel density was significantly lower in comparison to eyes without MvD. Macular GCIP thickness were significantly lower in optic neuritis eyes with MvD 6 months after diagnosis compared to those eyes without MvD. Logistic regression analyses also showed that the presence of MvD in optic neuritis was significantly associated with reduced GCIP at 6 months. To the best of our knowledge, the present study is the first to identify MvD in optic neuritis and characterize its association with longitudinal RNFL and GCIP thinning.

Optic neuritis, or inflammation of the optic nerves, is frequently associated with MS [19]. In MS, it is pathologically similar to brain lesions, characterized by inflammatory demyelination involving CD8+ T-cell activation [20]. Perivascular deposits of activated complement proteins and immunoglobulins are also typically found in affected lesions [21]. Developments in the past decade have revealed numerous other pathogenic mechanisms. For instance, the emergence of protein conformation-dependent assays found autoantibodies against MOG in central nervous system (CNS) [22], which can cause inflammatory demyelination to result in a myriad of CNS syndromes, including optic neuritis [23]. The lesions are characterized by perivascular infiltration by MOG-laden macrophages and CD4+ T cells [14, 21]. Autoantibodies against AQP4 water channels on astrocytes were also found, and they are associated with NMOSD, another autoimmune CNS inflammatory disorder, characterized by recurrent attacks of severe optic neuritis and/or myelitis [24]. Other causes of optic neuritis include infection, granulomatous disease, and paraneoplastic disorder [25–27].

The presence of MvD in optic neuritis as demonstrated in this study is another example that MvD is not specific to glaucoma, and that the phenomenon may be encountered in a setting that involves damage to the optic nerve. The mechanism of development of MvD in optic neuritis, however, is unclear. In NAION, MvD has shown a strong spatial correspondence with RNFL defects, which has led the investigators to conclude that MvD in NAION develops secondary to RNFL defect [9]. MvD was also demonstrated in CON, a disease in which optic nerves are damaged irrelevant of peripapillary vascular insufficiency [10]. Similarly, authors concluded that MvD was present as a byproduct of retinal ganglion cell death. In glaucoma, however, controversy is still ongoing. Some posit that MvD appears secondary to reduced metabolic demand of damaged axons, just like other optic neuropathies based on numerous investigations that showed proportional enlargement of MvD with disease progression [28]. Others have contended that MvD marks disruption in the vascular supply to the prelaminar region of the ONH [4]. These arguments are derived in part from anatomical features of peripapillary choroidal vessels which have been shown to provide branches to the lamina cribrosa and prelaminar region within the ONH [2]. Clinical investigations have also demonstrated association between MvD and faster RNFL thinning [5, 6], and VF progression [29]. Optic neuritis, the topic of our study, is a disease with complex mechanisms involving both axoplasmic flow stasis and inflammation, and the results of our analyses showed that the development and significance of MvD in this disease may be just as complex.

For one, our findings on reduction of peripapillary retinal vessel density in the temporal quadrant, the location that corresponded to the site at which MvD was most frequently found, may be interpreted as a phenomenon secondary to damaged axons in optic neuritis. Such spatial correspondence has been interpreted as evidence supporting the notion of secondary change. As mentioned previously, a similar finding was reported by Lee et al. in CON. Peripapillary vessel density of the CON eyes with MvD was significantly lower in the temporal quadrant in comparison to CON eyes without MvD [10]. In this study, MvD, when present in CON, was only detected in the temporal quadrant. In NAION, a strong correlation was found to exist between the distribution of MvD and RNFL defects [9]. Another study previously found that most significant perfusion reduction was noted in the temporal peripapillary quadrant in NAION, and the investigators concluded that the reduction in the temporal quadrant may be regression of superficial vessels in response to damage in the watershed zone [30]. In MS-associated optic neuritis, the inflammation was found to leave axonal damage with an inclination towards the temporal quadrant [31]. Taken together, decreased vessel density of the temporal peripapillary area corresponding to the location of MvD in optic neuritis may be a result of the damage to the watershed zone and resultant decrease in vessel density. We acknowledge that the spatial correlation between MvD and RNFL thickness was less obvious in optic neuritis as our analyses showed that the temporal RNFL of eyes with MvD was not significantly thinner than eyes without MvD. However, it is difficult to disregard the increase in RNFL thickness in the early phases of optic neuritis due to axoplasmic flow stasis, and it is possible that any correlation, if at all present, was masked [32]. In fact, previous reports have claimed that RNFL thinning continues during the first 6 months following an acute attack of optic neuritis [33, 34]. Further studies are necessary to prove the relationship between vascular damage in optic neuritis and the development of MvD.

The association between MvD and macular GCIP, however, may be interpreted either way. Our analyses showed that the GCIP thickness at initial presentation was not significantly different depending on the presence of MvD. At 6 months of follow-up, however, the GCIP thickness of patients with MvD was significantly decreased in comparison to patients without MvD. Acute inflammatory process of optic neuritis is believed to result in significant loss of axons and subsequent loss of retinal ganglion cells through retrograde degeneration [35, 36]. In contrast to RNFL thickness, which fluctuates during the course of optic neuritis, GCIP is minimally affected by axoplasmic flow stasis and is considered a reliable indicator of neurodegeneration from optic neuritis even in early stages [37, 38]. Permanent loss of retinal ganglion cells in optic neuritis in the form of GCIP thinning is believed to begin within 1 month of attack, and progress most rapidly during the 1^st^ month [32, 39, 40]. If MvD were a product of focal microvasculature loss secondary to preceding ganglion cell damage, initial lack of difference in GCIP thickness between patients with and without MvD is counterintuitive. If MvD is instead taken as a sign of choroidal vascular insufficiency, as shown by Jo et al. in glaucoma [4], it is not entirely unreasonable to assume that decreased GCIP thickness in all sectors at 6 months in the MvD+ group is the loss of ganglion cells from more severely impaired perfusion. Whether choroidal vascular insufficiency, if present in optic neuritis, is a contributing factor in the development of the disease or a product of extensive damage from inflammation needs to be studied further [41].

Irrespective of the mechanism of MvD in optic neuritis, its presence did not affect the functional outcome in the form of VF defect based on the results of our analyses. The MD, PSD and VFI of the patients with MvD were on average worse than those without MvD from initial presentation to the last follow-up at 6 months, but the difference was not significant. These results were somewhat unexpected because a number of previous reports has shown that the extent of neuronal loss in macular GCIP correlated with visual function [42, 43]. Studies in open-angle glaucoma have also shown that eyes with MvD tended to show faster VF progression, especially in the central region [29]. With regards to the results of the present study, we propose the following explanations. First, redundancy of neural network in macula might have compensated for damaged retinal ganglion cells, resulting in visual field parameters that do not reflect the amount of axonal loss [8]. Studies show that a significant proportion of optic neuritis patients, including those who sustain notable neuroaxonal damage, recover high-contrast acuity [44]. Second, current modalities that test vision performance may not be sensitive enough to detect the slight difference in visual function between the two groups. A lack of correlation between GCIP thickness and visual field tests has been previously reported in another study on optic neuritis, and in this study, too, the authors suspected that the a more sensitive means to evaluate vision performance might be necessary to uncover any correlation [39]. Third, we speculate that a significant difference might have been noted if the VF test results were collected for longer than 6 months. Reports on the relationship between RNFL and/or GCIP thickness and VF parameters have previously noted that a time lag exists between initial nerve fiber damage and resultant VF defect [45]. Fourth, the underlying etiology for the optic neuritis may have affected the visual outcome. For instance, patients with NMOSD were found to show worse visual outcomes after optic neuritis in comparison to those with MOGAD or MS [46]. Lastly, the difference might have been significant if the sample size were bigger. In a clinical setting, the detection of peripapillary choroidal MvD in eyes with previous optic neuritis may help identify those who are more likely to show extensive GCIP loss. However, further investigations may be necessary to determine whether MvD may also serve as an indicator of greater functional deterioration.

The present study has some limitations. First, the small sample size might have affected the accuracy of statistical analyses. Second, although it did not reach statistical significance, the follow-up duration between the 2 groups were considerably different and it may have affected the results. Third, projection artifacts of superficial vessels may have created false images of vascularity in the deep retinal layers, resulting in false negative detection of MvD. Fourth, the study population included optic neuritis of mixed etiology, such as MS, NMOSD with anti-AQP4 antibody or MOGAD with anti-MOG antibody. The population also included those with previous attacks of optic neuritis. Several studies have highlighted differences between anti-AQP4 antibody and anti-MOG antibody-positive optic neuritis [47, 48]. RNFL and GCIP thinning has also been reported in MS patients including eyes without a history of optic neuritis [49]. However, there are reports that the course of macular inner retinal layer atrophy was reported to be similar between MS and NMOSD [50–52]. Furthermore, both MvD+ and MvD- groups were comprised of comparable proportions of MS, anti-AQP4 antibody and anti-MOG antibody-positive optic neuritis as well as recurrences, and we believe that if the difference in etiology had any effect, they would have canceled out and resulted in minimal bias. Lastly, swelling of optic discs at initial presentation made detection of MvD difficult in many cases, so MvD was determined at different time points for each patient (3.4±3.0 months after initial presentation). Hence, we were unable to ascertain any causal relationships between MvD and vessel density as well as thicknesses of RNFL and GCIP. However, we believe that our results clearly indicate an association between MvD and reduced GCIP thickness at 6 months, when an episode of optic neuritis is generally considered to be concluded.

## Conclusion

In conclusion, the present study identified peripapillary choroidal microvasculature in patients with optic neuritis. The microvasculature dropout was most frequently found in the temporal quadrant, accompanied by a localized decrease in the superficial retinal vessel density. Patients who displayed MvD showed thinner GCIP in all macular sectors at 6 months follow-up. The presence of MvD in optic neuritis was associated with thinner GCIP at 6 months. Further studies are necessary to elucidate the pathogenesis of MvD in optic neuritis and its role in the development and progression of the disease.

## Supporting information

S1 Table Comparison of retinal vessel density, and initial RNFL and GCIP thicknesses between optic neuritis eyes with and without choroidal MvD. (DOCX)

S2 Table Comparisons of follow-up VF parameters between optic neuritis eyes with and without choroidal MvD. (DOCX)
