# Supplementary material for: Peripapillary choroidal microvasculature dropout is associated with poor prognosis in optic neuritis
Source: PLoS One. 2023 Apr 27;18(4):e0285017. doi: 10.1371/journal.pone.0285017 (PMC10138827; doi:10.1371/journal.pone.0285017)
Supplement: S1 Table — (DOCX) [file pone.0285017.s001.docx]

**Supplementary Table 1.** Comparison of retinal vessel density, and initial RNFL and GCIPL thicknesses between optic neuritis eyes with and without choroidal MvD

|  |  |  | MvD+ | MvD- | P |
| --- | --- | --- | --- | --- | --- |
|  |  |  | (n=20) | (n=28) |  |
| Peripapillary retinal vessel density, mm^-1^ | | | |  |  |
|  | Global | | 16.1±3.8 | 17.6±1.8 | 0.185^†^ |
|  | Inner | |  |  |  |
|  |  | Superior | 17.2±3.1 | 17.7±2.2 | 0.933^†^ |
|  |  | Temporal | 13.4±4.9 | 16.9±4.1 | **0.012^†^** |
|  |  | Inferior | 16.3±3.3 | 17.6±2.6 | 0.079^†^ |
|  |  | Nasal | 16.2±4.3 | 17.5±2.6 | 0.281^†^ |
|  | Outer | |  |  |  |
|  |  | Superior | 17.2±3.8 | 17.5±2.5 | 0.722^†^ |
|  |  | Temporal | 16.3±4.4 | 21.9±19.5 | 0.096^†^ |
|  |  | Inferior | 16.9±4.7 | 18.1±2.7 | 0.544^†^ |
|  |  | Nasal | 15.8±4.5 | 17.3±2.0 | 0.306^†^ |
|  | Signal strength | | 8.8±1.4 | 9.2±1.0 | 0.592^†^ |
| Macular retinal vessel density, mm^-1^ | | | |  |  |
|  | Global | | 15.8±3.6 | 17.2±2.2 | 0.114^†^ |
|  | Inner | |  |  |  |
|  |  | Superior | 15.8±4.4 | 17.4±2.2 | 0.484^†^ |
|  |  | Temporal | 15.3±4.6 | 16.9±2.9 | 0.287^†^ |
|  |  | Inferior | 16.1±4.5 | 17.0±2.9 | 0.983^†^ |
|  |  | Nasal | 16.4±4.1 | 17.3±2.4 | 0.723^†^ |
|  | Outer | |  |  |  |
|  |  | Superior | 15.6±3.7 | 17.3±2.3 | 0.100^†^ |
|  |  | Temporal | 14.7±4.1 | 16.2±2.6 | 0.287^†^ |
|  |  | Inferior | 16.0±3.7 | 17.7±2.4 | 0.105^†^ |
|  |  | Nasal | 17.8±3.4 | 18.9±2.3 | 0.199^†^ |
|  | Signal strength | | 8.5±0.4 | 9.2±1.2 | 0.174^†^ |
| RNFLT, µm | | |  |  |  |
|  | Average | | 120.4±53.4 | 139.4±66.7 | 0.380^†^ |
|  | Superior | | 153.8±82.9 | 178.8±101.3 | 0.431^†^ |
|  | Temporal | | 93.2±49.6 | 98.0±57.1 | 0.612^†^ |
|  | Inferior | | 154.8±71.2 | 167.8±84.8 | 0.677^†^ |
|  | Nasal | | 80.2±25.2 | 109.4±66.7 | 0.285^†^ |
| GCIPLT, µm | | |  |  |  |
|  | Average | | 64.8±20.0 | 68.3±20.1 | 0.378^†^ |
|  | Superior | | 73.7±11.0 | 74.2±18.3 | 0.321^†^ |
|  | Superotemporal | | 75.1±10.3 | 73.3±17.4 | 0.588^†^ |
|  | Inferotemporal | | 72.8±14.1 | 73.8±17.1 | 0.406^†^ |
|  | Inferior | | 68.1±16.9 | 72.8±16.4 | 0.259^†^ |
|  | Inferonasal | | 73.3±14.4 | 75.9±17.5 | 0.169^†^ |
|  | Superonasal | | 74.3±13.6 | 75.4±18.6 | 0.204^†^ |

P-value <0.05 was considered statistically significant.

^*^P <0.05 by Student's t-test

^†^P<0.05 by Mann-Whitney U test

Abbreviations: RNFLT, retinal nerve fiber layer thickness; GCIPLT, ganglion cell-inner plexiform layer thickness; MvD, microvasculature dropout.
