# Supplementary material for: Peripapillary choroidal microvasculature dropout is associated with poor prognosis in optic neuritis
Source: PLoS One. 2023 Apr 27;18(4):e0285017. doi: 10.1371/journal.pone.0285017 (PMC10138827; doi:10.1371/journal.pone.0285017)
Supplement: S2 Table — (DOCX) [file pone.0285017.s002.docx]

**Supplementary Table 2.** Comparisons of follow-up VF parameters between optic neuritis eyes with and without choroidal MvD

|  | Visual field | | | | | | | | | | |
| --- | --- | --- | --- | --- | --- | --- | --- | --- | --- | --- | --- |
|  | MD (dB) | | |  | PSD (dB) | | |  | VFI (%) | | |
|  | MvD + | MvD - | P |  | MvD + | MvD - | P |  | MvD + | MvD - | P |
| Initial | -18.4±14.0 | -11.3±11.7 | 0.079^†^ |  | 4.6±3.1 | 5.7±4.3 | 0.378^†^ |  | 45.5±42.4 | 67.7±36.7 | 0.074^†^ |
| 1 month | -5.9±6.4 | -5.5±9.1 | 0.880^†^ |  | 3.8±3.5 | 3.3±3.5 | 0.668^†^ |  | 86.8±18.5 | 85.4±28.7 | 0.881^†^ |
| 3 months | -7.8±8.9 | -3.7±6.9 | 0.182^†^ |  | 3.7±3.1 | 3.2±3.3 | 0.662^†^ |  | 80.3±30.8 | 91.5±20.8 | 0.262^†^ |
| 6 months | 11.0±12.9 | -3.3±6.8 | 0.109^†^ |  | 2.8±2.5 | 2.7±1.8 | 0.891^†^ |  | 70.1±43.7 | 91.2±21.4 | 0.181^†^ |

P-value <0.05 was considered statistically significant.

^*^P <0.05 by Student's t-test

^†^P<0.05 by Mann-Whitney U test

Abbreviations: VF, visual field; MvD, microvasculature dropout; MD, mean deviation; PSD, pattern standard deviation; VFI, visual field index.
